# The Actual and Potential Aroma of Winemaking Grapes

**DOI:** 10.3390/biom9120818

**Published:** 2019-12-03

**Authors:** Vicente Ferreira, Ricardo Lopez

**Affiliations:** Laboratory for Aroma Analysis and Enology (LAAE), Department of Analytical Chemistry, Universidad de Zaragoza, Instituto Agroalimentario de Aragón (IA2) (UNIZAR-CITA), c/Pedro Cerbuna 12, 50009 Zaragoza, Spain; riclopez@unizar.es

**Keywords:** wine aging, glycosides, glutathione, mercaptans, terpenols, norisoprenoids, volatile phenols, vanillin

## Abstract

This review intends to rationalize the knowledge related to the aroma of grapes and to the aroma of wine with specific origin in molecules formed in grapes. The actual flavor of grapes is formed by the few free aroma molecules already found in the pulp and in the skin, plus by those aroma molecules quickly formed by enzymatic/catalytic reactions. The review covers key aroma components of aromatic grapes, raisins and raisinized grapes, and the aroma components responsible from green and vegetal notes. This knowledge is used to explain the flavor properties of neutral grapes. The aroma potential of grape is the consequence of five different systems/pools of specific aroma precursors that during fermentation and/or aging, release wine varietal aroma. In total, 27 relevant wine aroma compounds can be considered that proceed from grape specific precursors. Some of them are immediately formed during fermentation, while some others require long aging time to accumulate. Precursors are glycosides, glutathionyl and cysteinyl conjugates, and other non-volatile molecules.

## 1. Introduction

Winemaking grapes are quite unique fruits because they are grown not to be immediately consumed, but to make wine with them. From this point of view, the study of grape aroma cannot be limited to the pool of molecules directly responsible for the odors and flavors of grape and grape juice but has also to include those other chemical structures that, more or less directly, are specific precursors of relevant wine aroma molecules. This task began more than 40 years ago when French and Australian researchers reported the existence of glycosides and other precursors of linalool [1,2]. The task, however, has proved to be extremely difficult due to many factors, such as the chemical and biochemical complexity of the precursor systems, the long times required to see aging effects in wine, or the analytical challenges associated to obtaining reliable representations of wine sensory properties from analytical data [3,4]. The truth is that nowadays, in spite of many significant advances, there are not accurate criteria or accepted methods able to provide a reliable assessment of the grape aroma potential, except perhaps for aromatic varietals such as Muscat or Gewürztraminer. This is a bit of a paradox; the grape genome was untangled more than 10 years ago [5], but yet, we do not have a clear understanding of all the grape metabolites which will ultimately contribute to the aromatic sensory properties of wine.

**Figure 1:**
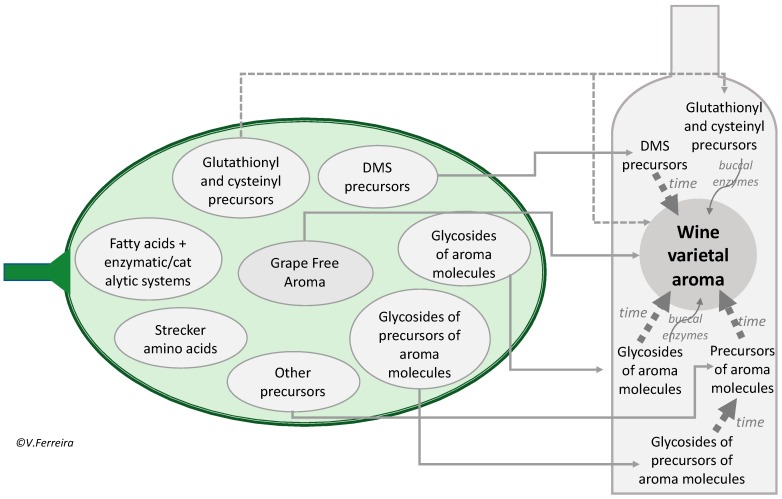
Scheme showing the main systems/pools in grape of specific precursors of aroma molecules and their involvement in the development of wine varietal aroma and flavor.

The reasons for this rather sluggish progress in linking grape molecular systems and wine odorants can be better understood with the help of the schema in Figure 1. The schema shows that grape contains at least seven different systems or pools of aroma precursors. Two out of the seven have relevance in grape but are not particularly important in wine aroma (the Strecker amino acid system and the fatty acid/peroxygenase system), while the other five play essential roles in the development of wine varietal aroma during wine aging, and/or in the development of wine flavor notes. If at the light of our present understanding, the different analytical strategies and concepts applied along the years for the study of grape aroma precursors are revisited, it will become evident that they provide information covering a rather limited fraction of wine varietal aroma. In fact, the general strategy followed to analyze grape glycosidic precursors deals with precursors belonging to just one or two out of the five pools. This is not to blame previous research, most of which was brilliantly carried out by pioneers, but to acknowledge the difficulties of the study, which with the limited analytical tools available in the 1980s, 1990s, and even the 2000s, hardly could have been done any better. 

The two first systematic approaches developed to study grape aroma precursors, which are yet the basis of the methods in use at present, were developed by Patrick Williams and coworkers in Australia [6] and by Ziya Gunata and coworkers in Montpellier [7]. In these approaches, grape glycosil aroma precursors are extracted from grape must or macerated grape solids with C18 or with XAD-2 polymeric sorbents, respectively. Much later, the use of more advanced polymeric sorbents providing a wider extraction of precursors was proposed [8], although as noted by Hampel et al., no sorbent was effective for all glycosides [9]. The glycosidic fractions are further hydrolyzed well by acid hydrolysis and enzymatic treatment [6], and well exclusively by enzymatic treatment [7]. 

The advantage of enzymatic treatment is that, in comparison to acid hydrolysis, it provides a relatively unbiased composition of the aglycones present in the extract, as far as the correct type of enzyme is used [9]. Under this approach the aroma of grape is divided into the free and the bound fractions [10,11]. Its major disadvantage is that, in many cases, the aglycone is not an odorant relevant for wine aroma, but an aroma-worthless volatile compound such as benzyl alcohol or an odorless precursor that only after a series of reactions, which can take a long time, will form the odorant. Attending to the scheme shown in Figure 1, enzymatic hydrolysis provides a useful estimate of wine aroma molecules derived from the pool of “glycosides of aroma molecules”, but not of those derived from the pool of “glycosides of precursors of aroma molecules” or from the other pools of precursors. Unfortunately, only some terpenols have direct glycosides, while important wine aroma molecules derived from norisoprenoids or grape phenols have not many direct glycosides. Consequently, enzymatic hydrolysis can assess the aroma potential of Muscat and other terpenol-related varietals, but not of “neutral varieties” [12]. 

For neutral varieties things are slightly more complicated, since the precursors of some relevant aroma molecules, such as norisoprenoids, require acid catalysis to undergo the chemical rearrangement processes through which the odorant is formed. Inevitably, this implies that labile aroma molecules, such as linalool and geraniol, will be degraded [9]. This problem is more evident in the many assays in which acid hydrolysis is carried out at high temperatures (100 °C). Under these conditions, as will be later detailed, there is a strong degradation of many relevant wine aroma molecules. Best results from the sensory point of view were obtained in the few studies in which acid hydrolysis was carried out at mild temperatures (45–50 °C). Only in these conditions the aroma hydrolysates obtained were able to induce significant sensory changes in wine [13,14]. However, some of the aroma descriptors developed during the hydrolysis, such as honey or tea [13], suggest that oxidation and thermal degradation processes are taking place under those conditions. These observations may question whether those hydrolysates are genuine representatives of wine varietal aroma and hence of grape potential aroma. 

A recently presented strategy tries to sort out these limitations by using a most powerful extraction strategy, carrying out the hydrolysis in strict anoxia and in the presence of grape polyphenols [15]. Grape polyphenols and most specific aroma precursors, except those of dimethyl sulfide (DMS), are coextracted from dearomatized “mistellas” and reconstituted in synthetic wine. Under these conditions, hydrolysates obtained after 24 h display sensory profiles congruent with unoxidized wine odor nuances and specific for the grape variety (Alegre et al., in preparation). The approach is promising, yet requires proper validation.

In the present review we will make a distinction between the actual and the potential aromas of grapes, even if in many instances the boundaries between both categories are relatively blurred. Actual grape aroma integrates those aroma molecules and chemical systems responsible for the aromatic sensory properties (odor and flavor) of grapes and grape juices. On the other hand, potential grape aroma refers to the different grape molecules and grape chemical systems that are specific precursors of relevant wine odorants.

## 2. The Actual Aroma of Grapes and Musts

**Figure 2:**
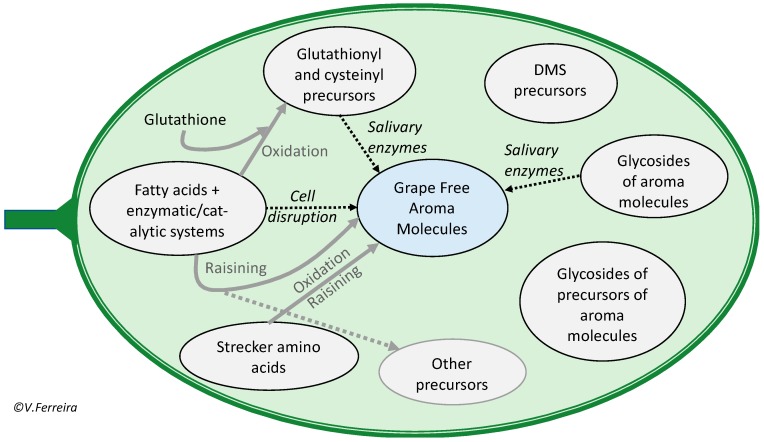
Scheme showing the different aroma precursor systems/pools in grape and their relationship with the fraction of free aroma molecules which will ultimately be responsible for the odor and flavor of grapes and musts.

The concept of actual aroma includes not only the aroma molecules found as free forms in the grape or must, but also those others formed in the short time span in which grapes of grape juices are kept in the mouth during mastication and salivation. This can be better understood with the help of the scheme shown in Figure 2. In the figure, the precursor systems able to quickly release free aroma molecules are linked by discontinuous arrows to the “grape free aroma molecules pool”. 

In common with many fruits, the actual aroma of grapes involves compounds in three related categories:

1. Free aroma, which refers to the aroma molecules found as such in the pulp and skin of the fruit, the grape in our case;
2. Aroma molecules formed by nearly instantaneous enzymatic/catalytical processes triggered during the disruption of fruit tissues [16,17];
3. Aroma molecules formed in the buccal cavity by the action of salivary or bacterial enzymes [18,19,20].

Compounds in the second category include a number of aldehydes, ketones and alcohols formed by peroxidation of fatty acids. Numerically the most abundant are compounds with six carbon atoms, so that compounds in this category are often named as C6-compounds [21,22]. It should be noted, however, that some powerful aroma compounds with a different number of carbon atoms can be also formed through this way, such as E-2-nonenal [23] or (E,Z)-2,6-nonadienal [24,25]. These powerful aroma compounds have much smaller odor thresholds, so that some of the green odors usually attributed to C6 aldehydes and alcohols could be in fact be caused by C9 aromas. 

Compounds in the third category derive from two different types of precursors. It has been demonstrated that glutathionyl and cysteinyl precursors, which are odorless cysteine-S-conjugates, can release the aromatic thiol by the action of buccal microbiota [18]. The release takes 20–30 s and can induce a perception lasting for up to 3 min, which supports the idea that these precursors can have an outstanding role in the persistence of grape and wine aroma. In the case of glycosidic precursors of aroma molecules, it has been demonstrated that oral bacteria are able to hydrolyze glycosidic precursors, releasing an array of volatiles [19]. In the particular case of glycoconjugates of the volatile phenols derived from smoke exposure, it was demonstrated that enzymes in saliva are able to release enough volatiles to create a sensory perception [26]. In the case of glycoconjugates extracted from Gewürztraminer grapes, sensory effects in the mouth were only evident when tested at 5-times wine concentration and in the absence of wine volatiles, which may call into question the sensory relevance of the aroma volatiles released from those glycosides in the mouth [20]. However, all these in-mouth effects are highly variable between individuals, so that for some sensitive individuals they may have an effect. Additionally, a recent report [27] has revealed that glycosides extracted from the grape marc added to the must produce wines with longer aftertaste. This observation does not unequivocally demonstrate the role of glycosidic precursors in aftertaste but supports their importance on wine flavor. 

In the case of grapes, the free aroma fraction is very small in most varieties, in agreement with the fact that most of them display weak and rather neutral odors and flavors. This should not be a surprise, since grapes are fruits extremely rich in water and do not contain special cellular or vacuolar structures in which nonpolar molecules such as aroma compounds can be safely stored. Hydrophobic molecules, including many aroma components, are stabilized in the pulp and skin by forming covalent bonds with polar molecules, such as sugars or amino acids, constituting fractions of specific aroma precursors which will be extensively discussed later on. 

In the present section we will focus on the aroma molecules which can be found as free molecules in grapes or musts and which are likely contributors of sensory notes. The section will be divided into four subsections. The first one addresses the aroma molecules of those types of grapes showing clear and distinctive aromas, such as Muscat, Gewurztraminer and some hybrids between *Vitis vinifera* and *labruscana*. The second subsection summarizes the knowledge about the aroma molecules of raisins. The third subsection considers aroma molecules responsible for green, herbaceous, and vegetal aroma, many of which form a kind of common background in grapes of all types. The fourth and last subsection will briefly discuss about the aroma molecules responsible for the aroma characteristics of neutral grapes.

### 2.1. Key Aroma Compounds of Aromatic Grapes

Among *Vitis vinifera* grape varieties, only those of the Muscat group have distinctive aroma and flavor [28]. These grapes contain important amounts of terpenols at levels above the odor threshold, as detailed in Table 1. The most important aroma compounds are linalool and geraniol, although those grapes also contain important levels of citral, citronellol, nerol, and α-terpineol. Another component, which attending to recent reports can be present at sensorily relevant levels, is geranic acid [29,30,31]. Muscat grapes can contain more than 5 mg/kg of these aroma compounds, in clear contrast to non-Muscat varieties which contain in general less than 0.5 mg/kg of these aroma compounds. Another relevant terpenic aroma compound is (Z)-rose oxide, which is responsible for the litchi-like or rose-like characteristic aroma of Gewürztraminer wines [32,33]. Rose oxide is a powerful aroma compound with an odor threshold one order of magnitude smaller than that of linalool [34]. It has been quantified in grapes from the Traminer family at 18 μg/L [35]. It has been recently found also in Muscat grapes [36] and a recent report even suggests that the intensity of Muscat aroma in grapes is strongly correlated to the presence of this molecule [37]. Its aromatic relevance in some aromatic grapes could have been underestimated simply because this molecule has been quantified in a reduced number of cases. Semiquantitative data provided by a recent report suggest that this aroma compound could be in fact relevant in the aroma profile not only of Gewurztraminer and Muscat, but also in Traminette and even in Riesling [38]. 

Among non *Vitis vinifera* cultivars there are some varieties known by their specific aroma. One of them is *Vitis labruscana* Bailey cv. Concord which contains at least four different aroma molecules at sensory-relevant levels. These are o-aminoacetophenone, methylfuraneol, methyl anthranilate, and furaneol [39,40]. Two of them, methyl anthranilate and o-aminoacetophenone, are involved in the characteristic “foxy” aroma of the variety (see Table 1). Remarkably, methyl anthranilate was identified as early as 1926 [41], while o-aminoacetophenone was identified in the 1980s [42]. Methyl anthranilate has been identified as one of the aroma components able to attract flies [43]. For its part, o-aminoacetophenone can eventually also develop in wines of *Vitis vinifera* varieties (mostly of German origin) where it causes a defect known as “untypical aging note” [44]. Furaneol (2,5-dimethyl-4-hydroxy-3(2H)-furanone) has also been identified as key odorant of muscadine (*Vitis rotundifolia* Michx), together with o-aminoacetophenone [45,46]. The potency and particular sensory characteristics of these aroma compounds make it so that those grapes are much appreciated as table grapes and also for making aromatic grape juice, but they are regarded as nonappropriate for making wine. In a recent paper, Wu et al. [29] study the aroma composition of 20 table grapes, 12 of which are hybrids between *V. vinifera* and *V. labrusca*. Interestingly, five of the hybrids showed strawberry aroma and four others foxy aroma, which suggests that the former contain large amounts of furaneol and of methylfuraneol, while the latter may contain methyl anthranilate and o-aminoacetophenone. Unfortunately, and this is a limitation of most recent studies carried out on grapes, all these polar and not very volatile aroma compounds cannot be easily determined by headspace solid phase microextraction (HS-SPME), which has become a kind of standard technique for the analysis of grape aroma. This explains the controversy about the implication of ethyl esters on the strawberry aroma of some of those grapes [29,47] and should warn about the risk of extracting conclusions about the sensory implications of analytical data when known essential aroma compounds have not been quantified: even if the profile of the volatiles quantified by HS-SPME is enough to obtain a highly satisfactory varietal differentiation, this does not mean that the varietal aroma profile is perfectly defined.

Some of these compounds can be also present, albeit at much smaller levels, in grapes from neutral varieties. For instance, furaneol was proposed time ago as a potential marker for the detection of forbidden hybrids (*Vitis vinifera* × non-vinifera) for making wine [53]. Furaneol can be present at levels above 1 mg/kg in non-viniferas, while it rarely will reach 0.05 mg/kg in *vinifera* wines [54]. 

Recent and quite extensive reports from Chinese researchers have confirmed that some table grapes contain a range of ethyl esters at concentrations above their thresholds [29,30,31,47]. These aroma compounds are found mainly as free compounds in the pulp and, in terms of odor activity values (OAVs), can amount to a relevant fraction of the odorants present in the grape. This fraction seems to be particularly high in “foxy” aroma grapes derived from *V. labruscana* [29] and also in some unfamiliar table-grapes [30]. For instance, in the cultivar “Honey Black”, these compounds account for more than 70% of the total OAV measured by the researchers. It is not clear, however, whether this aromatic power translates into specific aroma nuances. Ethyl esters are relatively ubiquitous aroma compounds and are normal constituents of the aroma of many fruits, so that they will likely contribute to generic fruity aroma nuances to grape flavor. 

### 2.2. Key Aroma Compounds of Raisins and of “Raisinized” Grapes

Another type of grapes with intense and specific aroma and flavors are raisins, which are grapes naturally dried under the sun or by different artificial means. Some raisins are used to make dessert wines, such as Pedro Ximenez, and are, therefore, genuine winemaking grapes. Many other raisins are produced to be directly consumed as sweet grapes and confectionery ingredients. Their aroma composition is, however, of general interest for the wine industry, since winemaking grapes can undergo naturally spontaneous drying processes on the vine (raisining, as indicated in Figure 2) as the consequence of different maturation problems. As those problems become more frequent due to climate change, unwanted raisining will be an emerging problem in many vine growing areas [55]. In the event these raisinized grapes are fermented together with healthy grapes, the wine will eventually develop raisin and prune notes. 

Raisins can contain different groups of key aroma compounds [23,56,57,58], which explains the high diversity of aroma nuances observed between different types of raisins and also supports the general complexity of raisin aroma. Leaving aside key terpenic odorants, such as linalool, geraniol, and rose oxide, which come directly from the fresh grape in the frequent case in which the raisins are made of aromatic grapes (Muscat and derivatives, Traminer and derivatives, Pedro Ximenez) [23], raisins can contain relevant odorants or groups of odorants produced or accumulated well during the own raisining process, during the last stages of grape maturation, and even during the storage of raisins. 

The first aroma compound particularly relevant in raisins is β-damascenone, which seems to be a quite ubiquitous and key aroma component of many sun-dried grapes [23,57] and of the wines made with them [59]. β-Damascenone is a norisoprenoid derived from the degradation of carotenoids. It has a quite low odor threshold, close to the ng/L, and an odor reminding of prunes or overmatured plums. As will be later discussed, this molecule plays also a relevant role in the flavor of neutral grapes and in the sensory properties of wines. Its structure and odor properties, together with those of other important aroma compounds from the same family, can be seen in Table 2. Different studies confirm that β-damascenone tends to accumulate in grapes in the last periods of maturation [60,61,62,63], particularly in the case of late season berry dehydration (or raisining) [64,65], during the storage of the raisins [58], or even during the aging of wines made with raisins [66]. Its levels, however, have no clear relationship with sun exposure on the vine [67,68]. β-Damascenone plays an outstanding role in the fruity aroma characteristics of wine. At low concentrations it acts as aroma enhancer [69] but at levels above 2–3 μg/L it can induce the perception of overmatured fruit, particularly if methional is also present [70].

The second group of powerful aroma compounds likely formed during grape dehydration are Strecker aldehydes derived from the Strecker degradation of amino acids. The most relevant from the aromatic point of view are phenylacetaldehyde (honey odor) and methional (raw potato odor), which are important aroma constituents of Pedro Ximenez wines made with sun-dried grapes [59]. Phenylacetaldehyde has been also found at levels well above its threshold in raisins [23,57]. The formation of these compounds can be particularly intense in the frequent case in which dehydration occurs after or during the attack of the fungus *Botrytis cinerea* [77,78,79], which explains the high levels of both compounds in wines from Sauternes. These compounds arise by the reaction of the amino acid precursor with a quinone or other α-dicarbonyl. In grapes, the major source of dicarbonyls is the quinones from oxidizing polyphenols. The oxidation can begin by photoactivation (normal raisining) or by enzymatic action, which will be particularly intense in the presence of the powerful phenol-oxidase from *Botrytis* (laccase). Recent results suggest that the formation may take place after some time of the solar irradiation, since in a study of the effects of the storage on raisin aroma, phenylacetaldehyde strongly accumulated only after 12 months of storage of sun-dried raisins but not in air-dried raisins [58]. These compounds are relatively difficult to analyze because of their high activity towards many chromatographic phases and because of the adducts they form with SO_2_. This explains why many reports fail in their detection, particularly in the case of methional, so that their importance may be underestimated. 

The third group of aroma components of raisins is formed by two odorous lactones derived from grape lipids, namely γ-nonalactone and massoia lactone. γ-Nonalactone is a well-known wine component [72] of coconut aroma whose levels in wine were first tentatively related to the development of prune character by Pons et al. [80]. The contribution to dry-fruit aroma has been recently shown to happen by perceptual interaction with furaneol and homofuraneol [81]. Its levels are increased in wines made from grapes affected by *Botrytis* [78,79], in late harvest wines [82], and in wines made from raisinized grapes [64]. Remarkably, γ-nonalactone is also a constituent of raisins [57]; its level and fate much depends on the type of grape, its pretreatment, time of storage and packaging material [58,83]. Massoia lactone (5,6-dihydro-6-pentyl-2H-pyran-2-one) has been recently identified as key aroma component in musts showing clear over-ripe characters of cooked plums and dried figs [84]. Both components, γ-nonalactone and massoia lactone, have been found at higher levels in wines made from partially dehydrated (raisinized) Shiraz grapes [65]. Massoia lactone has been also identified in the hydrolysates of phenolic and aromatic fractions (PAFs) extracted from grapes [15].

The fourth group of relevant aroma compounds formed during grape dehydration are some pyrazines with roasted aromas derived from Maillard reactions between sugars and amino acids. Wang et al. [23,57] identified at sensory-relevant levels 3-ethyl-2,5-dimethyl pyrazine and 2,6-diethylpyrazine. Both compounds were found to increase with storage of raisins [58].

Finally, and in common with any kind of grapes, raisins contain a relatively wide array of aldehydes and alcohols derived from the oxidation of grape fatty acids (FAOs). According to Wang et al. [23,57], pentanal, hexanal, heptanal, nonanal, decanal, (E)-2-hexenal, (E)-2-heptenal, (E)-2-octenal, (E)-2-nonenal, and 1-octen-3-ol can be found at levels above sensory thresholds.

The effects of dehydration on aroma composition are strongly dependent on many factors poorly controlled, such as the previous physiological state of the grape or the environmental conditions. Such variability has been observed for terpenols [85]. There are reports in which no changes in these compounds are observed during dehydration [86], others in which dramatic decreases were seen [87], and even others in which slight increases were measured [37,88]. A similar degree of diversity of patterns was also identified in the case of β-damascenone. Increased levels of this component, and also of γ-nonalactone [64] and of massoia lactone [65], have been observed and related to the prevalence of prune and fig character of the wines made with partially raisinized grapes [80]. In contrast, other studies have shown that shriveled grapes did not produce wines with higher β-damascenone content [89]. In the case of Strecker aldehydes, levels formed will be likely strongly related to the levels of the amino acid precursors (methionine and phenylalanine) present in the grape.

Regarding aldehydes and alcohols from FAOs, these compounds in general decrease during grape dehydration [56,86,88,90,91]. Such decreases may be attributed to a reduction in the lipoxygenase activity [86,90,91] of the raisinized grapes which cannot compensate for the general and continuous decrease of aldehydes by reaction with, among others, grape polyphenols. 

### 2.3. Aroma Compounds Responsible for Vegetal and Green Aroma and Flavors

There are two families of aroma compounds which play a role in the vegetal, herbaceous, and green–unripe characteristics of grapes, musts and, eventually, wine: alkylmethoxypyrazines along with aldehydes and alcohols derived from the oxidation of fatty acids, or fatty acid oxidation-derived odorants (FAOs). 

Alkylmethoxypyrazines are extremely powerful aroma molecules which accumulate in some grapes. They were first found in wines from Cabernet Sauvignon [92] and were further identified in Sauvignon Blanc juices and wines [93]. These compounds are 3-isobutyl-2-methoxypyrazine (IBMP), 3-secbutyl-2-methoxypyrazine (SBMP), and 3-isopropyl-2-methoxypyrazine (IPMP). Their properties are listed in Table 3. These compounds accumulate preferably in fruits grown under cool conditions and their levels decrease during ripening. They have been blamed for the specific green bell pepper character associated with Cabernet varieties, with a threshold for this character estimated to be just 15 ng/L [94]. Carmenere wines, which also belong to the Cabernet family, contain large amounts of these compounds too. Levels of IBMP were found to be strongly affected by climatic conditions and by vine genotype [95]. Temperatures during spring were found to be an important driver of green characters [96]. Levels of IBMP have been also positively related to altitude [97] and negatively related to light exposure, which limits accumulation but does not promote degradation [98]. Consequently, leaf removal significantly reduces accumulation of IBMP but only if it is carried out before veraison [99]. The relationship with nitrogen fertilization seems to be indirect, through the higher vigor [100]. Anecdotally, huge levels of IPMP can be induced by some foreign ladybeetles, causing great concern [101]. The levels of these compounds in wines from Spain and other southern countries are very low. It should be remarked, however, that strong negative correlations between the levels of these compounds—notably IBMP—and the different fruity and liquorice attributes of wines have been found in a recent work [102]. Such negative correlation would suggest that these compounds could be relevant suppressors at subthreshold level. 

The second family of compounds is formed by a relatively large number of aroma compounds, most of them aldehydes, derived from the oxidation of fatty acids or FAOs. Since quantitatively the most abundant were C6 alcohols and aldehydes, the family was first referred as the C6-family, however, some of the most powerful aroma compounds have nine carbon atoms, such as E-2-nonenal or (E,Z)-2,6-nonadienal. For instance, the most relevant aroma compound of Cabernet Sauvignon must, as assessed by aroma extract dilution analysis was (E,Z)-2,6-nonadienal [107]. Chemical structures and basic properties of these compounds are given in Table 4. This group of compounds derives from the enzymatic oxidation of fatty acids during must processing [22] and are well-known for the green odor of green leaves particularly evident in some teas [21]. The most powerful in aroma are the aldehydes, as usual, which have odor thresholds hundreds of times smaller than those of the corresponding alcohols. These aldehydes are surely responsible for the herbaceous note characteristics of some musts, particularly of those produced from unripe grapes. However, aldehydes are mostly eliminated during fermentation, in which they are enzymatically reduced to the corresponding alcohols. Consequently, the role of the family on the green and herbaceous (negative) aroma characteristics of wines has yet to be clearly demonstrated. FAO odorants decrease with maturity. Their levels are strongly related to grape variety [108] and also to the position in the bunch [109], being richer in the shoulder. 

The vegetal aromas of Cabernet Sauvignon and other wines are, however, much more complex and cannot be completely explained just by analyzing IPMP and IBMP [116], or C6-alcohols. While some works from Allen’s group initially reported a high correlation between the sensory vegetative aroma notes of Cabernet Sauvignon grapes grown in five sites of Sonoma and IBMP levels, more recent reports have not been able to find any correlation [116]. In fact, a comprehensive understanding of the green and unripe characters of wines remains a major challenge for wine science today. Preliminary reports from our group suggest that (a) C6-alcohols together with IBMP can impart herbaceous notes to red wine [117]; (b) the concerted action of hexanol, the major C6 alcohol, with dimethyl sulfide and methanethiol, opposed to the action of acetaldehyde and linear fatty acids, could be related to the vegetal character of wine [70]. 

There is also strong evidence demonstrating the implication of 1,8-cineole, a terpineol of eucalyptus odor, in the green and minty characters of wine. In many instances, the origin of this molecule is exogenous, coming from leaves of *Eucalyptus* trees [118] or from invasive plants, such as *Artemisia verlotiorum* [119]. Highest levels are related to the presence of the *Eucalyptus* leaves or of small quantities of the plant in the fermentation tanks, but the molecule can accumulate in the berry skin at sensorily relevant levels [120]. Additionally, recent evidence has shown that the molecule can be found in unripe berries of Cabernet Sauvignon and Merlot [119], contributing to the green perception via perceptual interaction with IBMP. A third formation route of 1,8-cineole in wine as product of the reaction of limonene and other terpenols has been also reported [66,121].

### 2.4. Compounds Responsible for the Flavor of Neutral Grapes

The subtle flavor of neutral grapes is the consequence of the presence of very small amounts of a relatively large list of aroma compounds. The list includes nearly all the aroma compounds described in the three previous subsections, the difference being that neutral grapes do not contain any odorant at the concentrations at which it can be regarded to act as impact aroma compound. In fact, studies performed on the aroma composition of neutral varietals, such as Grenache, Monastrell, Tempranillo, Aglianico, or Uva di Troia, using direct liquid–liquid extraction or solid phase extraction only find at quantifiable levels C6 compounds together with minor levels of some hydrocarbons, alcohols, ketones, esters, and terpenes [122,123,124,125]. Methods using SPME can more easily find other nonpolar volatiles, because of its intrinsic higher concentration power [126], but at the expense of losing the most polar and less volatile ones, such as furaneol or vanillin derivatives. 

Many neutral grapes contain low amounts of free furaneol, limonene, linalool, geraniol and other terpenols, β-damascenone, β-ionone and other norisoprenoids, and also of ethyl esters, such as ethyl butyrate, ethyl hexanoate, some volatile phenols, and vanillin derivatives. All these compounds, together with FAO derivatives, contribute concertedly to the subtle fruity flavor of neutral grapes. For instance, in one of the few works published about the gas chromatography-olfactometric (GCO) profiles of neutral grapes, the most relevant odorants were β-damascenone, β-ionone, ethyl hexanoate, ethyl octanoate, and different FAO derivatives (hexanal, decanal, and (E,Z)-2,6-nonadienal) [25]. With no impact aroma compound present, but with a relatively wide array of fruity–sweet–citric–flowery smelling aroma compounds present at low levels, there is a perceptual cooperation between all of them as described by Loscos et al. [127], whose outcome is a subtle sweet–fruity flavor. 

There is also some evidence that neutral grapes of specific varieties contain eventually sensorily-relevant levels of rotundone. Rotundone is a sesquiterpene that is also present in grapes and can give a peppery aroma to grapes and wines [128]. In certain varieties, like Shiraz or Duras, and under favorable agronomical conditions [129,130], rotundone can accumulate in the berry exocarp in levels in the order of 600 ng/kg [128]. The synthesis pathway of rotundone in grape is not clear, but α-guaiene has been proposed as a potential precursor [131]. During the red wine winemaking maceration process, rotundone is extracted and can reach levels well above its perception threshold of 16 ng/L [128,132]. This characteristic peppery aroma is usually perceived positively among wine consumers [133].

Following the idea of aromatic series proposed by different authors [29,31,125], it can be stated that the aroma of neutral grapes is the consequence of the concerted action of 25–30 aroma compounds, with aroma nuances classifiable into seven odor categories:

1. Fruity: ethyl isobutyrate, ethyl butyrate, ethyl 3-methylbutyrate, ethyl hexanoate, ethyl octanoate, and eventually others;
2. Jammy, very sweet fruit: furaneol, homofuraneol, β-damascenone, γ-nonalactone, and massoia lactone;
3. Sweet–floral: vanillin, ethyl vanillate, β-ionone, β-phenylethyl acetate, and phenylacetaldehyde;
4. Floral–citric aroma compounds: linalool, geraniol, limonene, nonanal, and eventually others;
5. Herbaceous: hexanal, (Z)-3-hexenal, (E)-2-hexenal, (Z)-3-hexenol, (E)-2-nonenal, (E,Z)-2,6-nonadienal;
6. Peppery: rotundone;
7. Unspecific: 3-methylbutanal, ethyl acetate, diacetyl.

## 3. Grape Potential Aroma: Specific Aroma Precursors

### 3.1. Specific vs. Unspecific Precursors

Grape specific aroma precursors are non-volatile and hence odorless molecules which may rend a specific odoriferous molecule by the hydrolysis of a chemical bond, by spontaneous chemical rearrangement, or by a combination of both mechanisms. Many grape and grape-derived wine aroma molecules have specific aroma precursors. Remarkably, some of them have a relatively complex pool of different “specific precursors”. This is common in nature; for instance, apples contain more than eight different non-volatile molecules which by hydrolysis and further chemical rearrangement lead to β-damascenone [134]. A higher level of complexity regarding the number and type of precursor molecules is found in grapes. Such a pool of molecules is the pool of β-damascenone precursors. Similarly, there is a pool of precursors for linalool, for geraniol, for (Z)-rose oxide, for β-ionone, for furaneol, for TDN, for 3-mercaptohexanol, and for many other relevant grape-derived wine aroma compounds. 

The word specific has an important meaning here. “Specific” means that the aroma compound will be formed by simple incubation of the pool of precursors extracted from grape at normal wine pH, or alternatively, by incubation in the presence of an enzyme. This definition deliberately excludes those precursor molecules which can be transformed into aroma compounds only by a complex metabolic action of yeast, bacteria or other micro-organisms. For instance, the amino acid isoleucine can be metabolized by *Saccharomyces* producing as byproducts isovaleric acid, isoamyl alcohol and isoamyl acetate. But isoleucine cannot be regarded as a specific precursor for these important aroma compounds, because their final levels are extraordinarily constrained by the metabolic requirements of yeast. In fact, yeast is able to produce all those compounds even if there is no isoleucine in the fermentation media. We rather should consider it as an unspecific precursor of the aroma molecule. This differentiation has a paramount importance for defining grape aroma potential. In general, wines made from grapes containing higher levels of specific precursors will develop higher levels of the aroma molecules derived from those precursors and/or will keep levels of those molecules for longer aging periods. 

### 3.2. Grape Aroma vs. Grape-Derived Wine Aroma

As was schematized in Figure 1, grapes contain seven relatively well differentiated chemical/biochemical aroma precursor systems. As discussed previously, two of the systems—the fatty acid/enzymatic system and the Strecker amino acid system—have a major role in the development of the actual aroma of grapes, but to the best of our knowledge, they seem to have a rather limited role as wine aroma precursors. Both systems will influence wine aroma insofar as they form grape aroma molecules or precursors of aroma molecules, which will eventually pass to wine, but the systems as such do not survive fermentation. This explains why if the grape has not suffered raisination or over-ripening, the wine, generally, will not develop prune and overmatured character. On the contrary, the five other systems or molecular pools will be transferred to wine with different degrees of change induced by fermentation and will release or produce the specific aroma molecules at different moments of the winemaking process.

The wine odorants for which there is more or less strong evidence about the implication of grape specific precursors in their formation are summarized in Table 5, Table 6 and Table 7. The list includes 27 compounds: four norisoprenoids, five terpenes, six volatile phenols, four vanillin derivatives, ethyl cinnamate, two ethyl esters, two lactones, furaneol, DMS, and three polyfunctional mercaptans. Some compounds in the list, such as polyfunctional mercaptans, DMS, linalool, rose oxide, or TDN, can reach odor-impact levels. Some others, such as volatile phenols or vanillin derivatives, rather exert a cooperative effect on wine aroma. Mint lactones, recently identified at low levels in red wines from Bordeaux [135], limonene and 1,4- and 1,8-cineol, as well as some megastigmatrienones, may also play a role in minty, balsamic, and tobacco notes [66], but evidence about their implication is yet weak. 

The tables summarize information relative to the presence of the odorants in hydrolysates obtained by enzymatic, harsh, or mild (long term) acid hydrolysis. This information is relevant to understand the genesis of the aroma compound and also to assess the relevance of the findings of the different reports. In some of the few studies using long term acid hydrolyses, there is additional information about the pattern of accumulation of the odorant with time. This information is crucial to understand the evolution of these aroma molecules during wine aging. As can be seen in Table 5, none of the four norisoprenoid odorants were present in enzymatic hydrolysates. Only in grapes kept frozen before the analysis, or in raisins, were these odorants found after enzymatic hydrolysis. In the case of terpenes (Table 5), volatile phenols, and vanillin derivatives (Table 6), enzymatic hydrolysis in general produced much higher levels than harsh acid hydrolysis. By contrast, most compounds are found at reasonable levels in hydrolysates obtained by long-term acid hydrolysis. 

Large differences between compounds are also found regarding the pattern of accumulation during aging. Linalool and geraniol reach maximal levels immediately after fermentation or after a short aging time, and afterwards their levels decay dramatically. β-Damascenone and β-ionone reach maximal levels also after a relatively short aging period, but their levels decay slowly or stay stable (Table 5). By contrast, TDN, TPB, and most volatile phenols and vanillin derivatives steadily increase during aging (Table 5 and Table 6). 4-Vinylphenol and 4-vinylguaiacol follow more complex evolutions with at least two maxima, likely because of the number of precursors they have and their chemical reactivity. The evolution with time of some relevant odorants, such as (Z)-rose oxide, geranic acid, or piperitone is mostly unknown.

Data summarized in the tables also reveal the existence of huge variabilities in the levels of most compounds, regarding variety, vintage, location, or maturity. While some differences may be attributed just to the different analytical methodologies followed by the researchers, some others truly reflect a large diversity. Differences between Muscat grapes and “neutral” grapes regarding levels of terpenols are known, as well as those of furaneol between hybrids and *Vitis vinifera* varieties. However, data in Table 6 suggest that differences in the levels of some volatile phenols and vanillin derivatives are well above the order of magnitude.

Finally, Table 7 contains some odorants for which the existence of precursors can be expected but has not been demonstrated. 

The following four sections deal with the different types of precursors responsible for all those odorants. The first section deals with glycosidic precursors, the second with other precursors, and the two last sections with glutathionyl and cysteinyl precursors and DMS precursors. 

### 3.3. Glycoconjugates as Aroma Precursors

Some good reviews on these questions have been recently published [154,155,156]. Glycoconjugation is a clever way to solubilize and fix nonpolar and volatile aroma molecules and it is very common in nature [157]. Many secondary metabolites of plants are glycoconjugated, and in fact, glycoconjugation can be considered a relatively common last step of plant secondary metabolism and seems to be a primary sedative mechanism used by plants to maintain metabolic homeostasis [158] and to detoxify from potentially toxic (lipophilic and/or reactive nucleophiles) molecules [159]. Glycoconjugation takes place by reaction between a reactive functional group and an “activated” sugar. Activated sugars are UDP-glucose, UDP-rhamnose, UDP-galactose, UDP-xyloxe and glucuronic acid, where UDP stands for uracil-diphosphate glucose. The reactive functional groups are -COOH, -NH_2_, -SH, and -OH, among others.

In the case of grapes, little is known about the real activities and selectivities of glycosyltransferases, but at least 240 different types of these enzymes are coded in the grape genome [160]. Although glycosides may be more easily handled and transported by plant transport systems, recent evidences suggest that grape aroma glycosides are integrally formed in the grape. 

**Figure 3:**
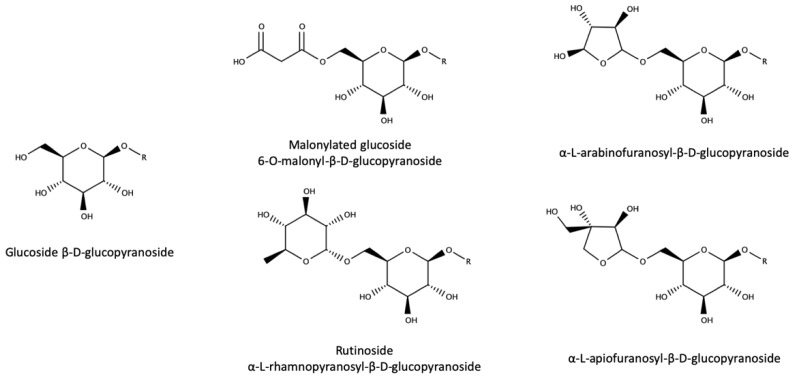
Sugar moieties of glycoside precursors. Adapted from [154].

Of course, major grape glycosides are those of flavonoids, phenolic acids, and anthocyanins, while aroma compounds represent quantitatively a quite modest fraction. In the case of aroma compounds, to date, all grape aroma-related derivatives have been found to be bound to a β-D-glucose, and such glucose can be further bound to malonic acid, arabinose, apiofuranose, or rhamnose to form the structures indicated in Figure 3.

Recently, two trisaccharides have been also tentatively identified in grape [161,162].

According to the type of aglycone, glycoconjugates in grapes can be broadly classified into the following categories:

1. Aliphatic alcohol derivatives;
2. Terpenes;
3. Norisoprenoids;
4. Benzenoids, which can be further subdivided into:
  - Benzyl and phenyl derivatives;
  - Volatile phenols;
  - Vanillins;
  - Ethyl cinnamate.
5. Miscellaneous compounds.

Aliphatic alcohol derivatives can be quantitatively important, but they are quite unimportant from the aromatic point of view. Compounds in this group, among others, include isoamyl alcohol, hexanol, (Z)-3-hexenol, (E)-2-hexenol, 1-octen-3-ol, heptanol, and octanol [138]. 

**Figure 4:**
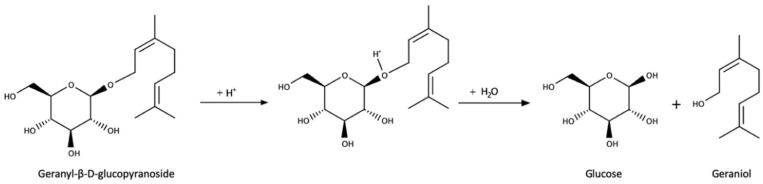
Release of geraniol via acid-catalyzed hydrolysis of the geranyl-β-D-glucopyranoside.

Terpenes include a quite complex array of terpenes in different oxidation states. The list includes several terpenic diols which will be presented in the next section, together with linalool, α-terpineol, nerol, geraniol, and several of their oxides, including c-rose oxide. The most important from the sensory point of view are the same four as in the free fraction, namely linalool, geraniol, c-rose oxide, and geranic acid. Note that some of these compounds will suffer chemical rearrangements at acidic pHs. Different reports have estimated that between 77% to 83% of the total terpenic content in Riesling grapes are present as glycosides [163,164,165]. Some of them, such as different hydroxylated forms of the main terpenols or of geranic acid, seem to be majorly or even exclusively found as glycosides [147]. From the quantitative point of view, major aglycones of terpenes in neutral varietals are those of geraniol (Figure 4), (Z)-8-hydroxy-linalool (or (2Z)-2,6-dimethylocta-2,7-diene-1,6-diol), and p-menthene-7,8-diol with account to more than 60% of the peak area, eventually followed by those of linalool and geranic acid and those of the (E)- and (Z)-pyran linalool oxides [137,140,150,166]. A glycoside precursor of 1,8-cineole, namely 2-exo-hydroxy-1,8-cineole, has been also identified in Falanghinna grapes [167]. 

There are a number of recent reports about the evolution of these precursors during grape maturation. Results show that the patterns of accumulation depend largely on the aroma compound [145], on the variety of grape [147], and on the vintage [146], which makes difficult to extract sound conclusions. In general, it can be said that glycosidic forms tend to increase with maturation following more regular accumulation patterns than free forms, which can show erratic patterns of evolution during maturation.

As summarized in Table 5, levels of linalool and geraniol are maximal in the wines immediately or shortly after fermentation, and levels decrease due to the poor stability of these molecules at wine pH. The pool of precursors which survived the fermentation seems to be essential for keeping the levels of these relevant aromas longer times [14,143].

Aglycones in the norisoprenoid family can be also extraordinarily complex and, not surprisingly, there are not aglycones representing the most relevant aroma compounds in this family, such as β-damascenone, β-ionone, TDN, or TPB. The major aglycones are 3-hydroxy-β-damascone, dihydro-β-ionone, and different ionols, particularly 3-oxo-a-ionol and vomifoliol [140,150]. This represent quite a nuisance, since the direct analysis of the aglycones (after careful enzymatic hydrolysis) or the direct HPLC-MS of the unaltered glycosidic precursors do not give clear information about the aroma potentiality of this important precursor fraction. 

There is large difference between the four major nor-isoprenic odorants regarding the pattern of accumulation during aging. β-Damascenone and β-ionone reach maximal levels soon and then remain stable or steadily decrease with aging. By contrast, TDN and TPB are formed much more slowly during aging, with levels steadily increasing, as indicated in Table 5. A recent report has shown that fermented samples form TDN faster than unfermented controls, which suggests that some of the first chemical reactions in the sequence required to form TDN from 3,6-dihydroxy-β-ionone, its main precursor [168], are accelerated by yeast [143]. Such a report also demonstrates that levels of TDN formed during aging can be modulated by yeast.

Within the group of benzenoids (Table 6) there are several subgroups of volatile compounds usually identified in the hydrolysates of grape precursor fractions [8,12,14,142,147].

Benzyl and phenyl derivatives include benzaldehyde, benzoic acid, benzyl alcohol, and 2-phenylethanol. In many neutral grape varieties these compounds, particularly the latter two, are the major constituents of the glycosidic aroma fraction [137,140,150]. This has some practical relevance since the contribution of these glycosides to wine flavor can be considered negligible. One the one hand, the odor thresholds of both odorants are relatively high, and on the other hand, 2-phenylethanol is a main secondary product of yeast metabolism, so that levels derived from grape glycosides represent a quantitatively marginal fraction. The consequence is that indirect measures for the aromatic potential of neutral grapes [169] may be not related to the true aromatic potential but just to the general secondary metabolic activity of the grape.

Volatile phenols, such as guaiacol, eugenol, isoeugenol, 2,6-dimethyoxyphenol, 4-vinylguaiacol, and 4-vinylphenol, are relevant components of the hydrolysates obtained from fractions of precursors extracted from grapes or wines, as detailed in Table 6. All or some of them tend to score high in the different GCO studies carried out on hydrolysates [139,142,170]. Reported levels of all these compounds have ranges of variation depending on vintage and varieties close to two orders of magnitude, as summarized in Table 6. These compounds cannot be determined by harsh acid hydrolysis, even though most of them accumulate steadily during aging. The case of vinylguaiacol and vinylphenol is particularly interesting. Both can be considered detrimental for wine quality if present at high levels [171]. As recently documented, they can be formed via yeast phenolic acid decarboxylases from phenolic acids and also by enzymatic or acid hydrolyses of their glycosides [143].

Vanillin and other related compounds are also formed from different precursors. Although the levels of these important aroma compounds derived from the grape cannot rival with levels released by some types of oak wood, grapes contain a large number of precursors able to release significant levels of these compounds. Vanillin is one of the odorants of acid hydrolysates which always scores very high by GCO [15,155,158,159]. In the enzymatic hydrolysates obtained from some varieties, such as those of skins from Uva di Troia [125] vanillin can be found at high levels (more than 360 μg/kg). Additionally, vanillin can be also formed by oxidation of 4-vinylguaiacol [172].

Ethyl cinnamate has been also found at minor levels in the hydrolysates of precursor fractions extracted from grapes (see Table 6). Since cinnamic acid was also identified as aglycone after enzymatic hydrolysis, the precursor should be a glycoside. A glycoside of cinnamic acid has been recently identified in wine made from Korean black raspberries [173]. 

Within the miscellaneous section (Table 7), the most relevant odorant is furaneol. Furaneol glucopyranoside has been recently identified and quantified in the must of Muscat Bailey A (*V. labrusca* (Bailey) × *V. vinifera* (Muscat Hamburg)) [174]. The gene encoding the UDP-glucose: furaneol glucosyltransferase was also determined [175]. The same authors were also able to quantify this precursor in different grape varieties and in the parental concord. Concentrations of the precursor were much higher in the *labrusca* and in the hybrids, but normal grapes also contain low amounts of this precursor. This aroma compound has been systematically identified by GC olfactometry in the hydrolyzed precursor fractions extracted from Grenache [142], Aragonez [139], Pinot Noir [170], or Tempranillo [15], and it has been found as aglycone released by enzymatic hydrolysis of the precursor fraction from Aglianico and Uva di Troia [125].

Finally, it should be noted that several authors have reported the presence of glycosides of some fatty acids at relatively large levels in the enzymatic hydrolysates of precursor fractions extracted from wines. For instance, isovaleric acid was found at 109 μg/L, butyric acid at 412 μg/L, hexanoic at 336 μg/L, and octanoic acid at 295 μg/L [150,152]. These amounts are just slightly smaller than those formed by yeast.

### 3.4. Other Precursors: Molecules Which by Chemical Rearrangement or Esterification Form the Aroma Molecule

The first type of molecules includes a series of polyols discovered more than 30 years ago which by chemical rearrangements induced by the acid hydrolysis at wine pH produce different aroma active terpenols [2]. 

**Figure 5:**
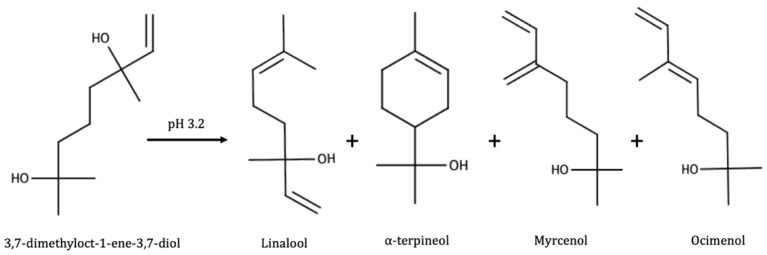
Polyol rearrangement at pH 3.2. Adapted from [2].

**Figure 6:**
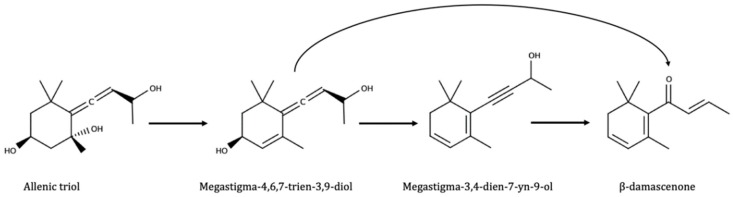
Formation of β-damascenone from allenic triol. Adapted from [181].

As shown in Figure 5, one of the diols (3,7-dimethyloct-1-ene-3,7-diol) rearranges to give linalool and α-terpineol. The other molecules are different terpenols of lesser olfactory importance such as myrcenol or ocimenol. The diols were also found to be present as glycosides [176]. Some C13-triols with a megastimagne structure were also further identified as potential precursors for some norisoprenoids such as vitispiranes and TDN [177]. At wine pH, these precursors can spontaneously form TDN, responsible for the kerosene–off odor developed by some wines during aging. Also a megastimagne structure, megastigm-5-en-7-yne-3,9-diol, was identified as precursor for β-damascenone [178]. This was later confirmed by synthesis of the pure molecule [179]. The dienyne derivative and the allenic diol, shown in Figure 6, were further identified in 2005 [180]. Both proceed from an allenic triol derived from the degradation of carotenoids such as neoxanthin [181].

Notably, Australian researchers have recently demonstrated that a ketone and a diketone derived from diol 5 can be transformed by the action of yeast in β-damascenone [182].

As previously mentioned, most of these molecules are also found as glycosides, which supposedly amount to a larger fraction of precursors.

Finally, in this section we should mention the two lactones and the two ethyl esters listed in Table 7: γ-decalactone and massoia lactone and ethyl cyclohexanoate and ethyl 4-methylpentanoate. The two lactones are primarily formed during grape dehydration, but since they accumulate in some wines or precursor fractions, it can be suggested that the corresponding γ-hydroxy or δ-hydroxy acids are present as precursors. As different glycosidic precursors of whisky lactones (γ-methyloctalactone) have been described in oak wood [183,184] the presence of some glycosides of the acids cannot be ruled out. In the case of the esters, the corresponding acids have been quantified in unfermented grape must [185].

### 3.5. S-Derivatives of Cysteine or Glutathione

Two recent reviews [186,187] have been published on cysteinyl or glutathionyl derivatives. Grapes contain some cysteinyl or glutathionyl derivatives which by hydrolysis of the S–C bond in the cysteine part can give some of the most powerful aroma molecules of wine and of nature in general. The aroma molecules are 4-methyl-4-mercaptopentan-2-one (4MMP), 3-mercaptohexanol (3MH), and 3-mercaptohexyl acetate (3MHA). The aroma properties of these relevant aroma compounds are summarized in the following Table 8 [187]:

There are at least three or four more other varietal polyfunctional mercaptans in wine with far less aromatic importance.

**Figure 7:**
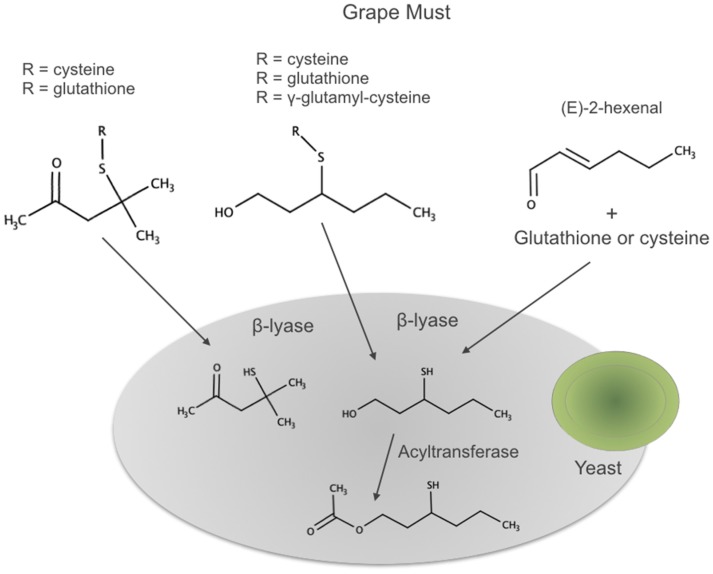
Biogenesis pathways of 4MMP, 3MH, and 3MHA. Adapted from [187].

All these aroma compounds are released by the action of β-lyase enzymes from yeasts from their specific precursors present in the grape must. The 3MHA requires, in addition, the acetylation of the alcohol 3MH by action of an acyltransferase also from yeast, as summarized in Figure 7.

Apart from the precursors described in Figure 7, very recent reports demonstrate also the existence of the glutathione precursor of 4-mercapto-4-methylpentan-2-ol [190] and of hexanal [191]. The first precursors discovered were the cysteinylated ones [192], and for over 10 years thiols were thought to be formed exclusively from cysteine conjugates. Glutathione precursors were identified much later and definitive evidence of their effective role as precursors of 3MH and 4MM4P was obtained only some years ago [193,194,195]. Recently, a glutamyl–cysteine dipeptide S-conjugate to 3MH has also been identified in must [196]. From the quantitative point of view, Glu–3MH precursor is the most concentrated, being present at levels between 8 and 35 times higher than those of the Cys–3MH precursor. In the case of MP, both can be at similar levels [197] (see Table 9).

The conjugated thiol precursors are produced in the grape and concentrations are highest in the skin [198]. Little is known, however, about their biosynthesis and about the factors determining their accumulation during grape maturation. Levels are varietal-dependent, being highest in Sauvignon Blanc and Verdejo and close to null in Malvasia del Lazio, and increase during maturation [190]. Levels are also related to picking time [199], being maximum at early morning and later decreasing during the day. Interesting changes in amino acid levels during the day have been also identified as a consequence of leaf photosynthesis [200].

As it is also suggested in the previous figure, there is an additional prefermentative pathway leading to the in situ formation of 3MH precursors during grape processing before fermentation. According to this pathway, 3MH precursors form once the berry is damaged by reaction between E-2-hexenal formed via enzymatic oxidation of grape fatty acids and cysteine or glutathione present in the must. The existence of such pathway resulted as evident by the observed paradox that hand-picked grapes from Sauvignon Blanc produced wines much less aromatic than those harvested by machine [201]. The relative importance of the two different “kinds” of precursors, those already present in the grape and those formed in situ during early grape processing, is not clear. Subileau et al. showed that in their conditions (E)-2-hexenal was not a major contributor [194], while different studies confirm that machine-harvested grapes contain higher levels, with excessive oxidation being detrimental [201,202]. The effects of maceration time and pressing have been also studied by several authors, mostly concluding that prolonged maceration times leaded to higher levels of precursors [203,204]. More recently, Larcher et al. demonstrated that oxygen at harvest was essential for increased levels of precursors [205]. The apparent contradictory observations could be related to the existence of several concurrent factors not yet well controlled in the experiments such as the E-2-hexenal formation rate of the grape (dependent on grape lipoxygenases, oxygen, and grape fatty acids) and the cysteine and glutathione availability of the must.

Cysteinyl and glutathionyl precursors are poorly metabolized by most yeasts, so that levels of the precursors in the final wines can be high [206], particularly if the must contains high levels of glutathione [15]. It should be noted that there is evidence, some old [142,207] and some new [15], suggesting that the powerful polyfunctional mercaptans could be also formed by acid hydrolysis of the precursors. The role of this pool of compounds to keep longer levels of these powerful aroma compounds should not be ruled out.

### 3.6. S-Methylmethionine and Other DMS Precursors

Dimethyl sulfide is a quite remarkable wine aroma compound. It has been repeatedly identified as a powerful aroma enhancer [117,208] and, more specifically, as a contributor to blackberry and blackcurrant aroma nuances of red wines [209].

This compound can be formed by spontaneous hydrolysis of different precursors (very fast at alkaline medium) [210], of which S-methylmethionine (vitamin U) has been identified as the most important [211]. There are nearly no other reports about the occurrence and factors affecting the levels of this precursor in grapes, although its level has been found to be related to water deficit of vines [212]. Vines with moderate water deficit have higher potential for this compound and the concomitant higher levels of yeast assimilable nitrogen contained in the musts from those vines seem additionally to avoid the destruction (metabolization) of the precursor during fermentation.

### 3.7. The Action of Fungus and Other Exogenous Factors on Grape Actual and Potential Aroma

Finally, the aroma of the must or grapes reaching the cellar can be strongly affected by the presence of fungus or by some other exogenous factors. Wines made from grapes affected by noble rot have higher levels of 3MH, furaneol, sotolon, methional, and phenylacetaldehyde [59,77,78], while wines made from grapes affected by uncontrolled fungal attacks can develop fungal odors. Some of them, at smaller levels, are of course also present in noble rot wines, such as 1-octen-3-ol [78]. The infection with *Botrytis cinerea* also changes some must enzymes with effect on aroma (esterase and β-glucosidase). 

Grapes affected by noble rot have also increased levels of cysteinyl precursors [213] and can have even an expanded number of this type of precursor [214], which helps explaining their particular aroma. 

Regarding negative odors related to fungal attacks, 3-octanone, 1-octen-3-one, (E)-2-octenol, 1-octen-3-ol, 2-methyl isoborneol, TCA, geosmin, TBA, and pentachloroanisole are usually targeted as responsible for off-odors [215]. The type and levels are related to the strain of fungus; 50% of *Botrytis cinerea* strains induce geosmin, one strain induces anisol [216]

Following the exposure of vineyards to forest or bushfires, the occurrence of the smoke taint has been detected repeatedly; one review has been published recently about this off-flavor in wine [217]. Volatile phenols, like phenol, guaiacol, and their derivatives, that usually appear in wines as a consequence of barrel toasting or contamination with *Brettanomyces* yeasts, are present in greater quantities in wines produced with grapes exposed to smoke [218]. The evidence that free run juice of smoked grapes had trace levels of volatile phenols, while the same juice after several days of maceration showed levels in the range of hundreds of μg/L, proved that volatile phenols were stored in the skin rather that in the pulp [219]. Several studies have confirmed that the accumulation of volatile phenols takes place in the form of different glycoconjugates [220,221,222]. The release of volatile phenols from their precursor forms takes place not only during fermentation via enzymatic hydrolysis, but also via acid hydrolysis during post-bottle aging [223].

## 4. Final Conclusions

Both grape aroma and grape-derived wine aroma are formed by a relatively large group of odorants belonging to different chemical and biochemical families. Only in the specific cases of aromatic grapes are there clear impact compounds or families of compounds defining the aroma profile. In neutral varieties, grape aroma profiles are rather the consequence of the presence of more than 20 odorants imparting at least seven different types of aroma nuances. In the case of wine, up to 27 relevant wine odorants have specific origin in grape molecules or specific aroma precursors. Those odorants have, however, a much larger aromatic diversity than that observed between grape odorants, introducing or contributing to many different wine odor nuances such as fruity, jammy, floral, citrus, phenolic, spicy, empyreumatic, or green, and hence contributing decisively to wine quality. 

Additionally, grape-derived wine aroma molecules accumulate in quite different time periods of winemaking; some of them are mostly released during fermentation, while some others accumulate only after long periods of aging. Within the first, remaining precursors in wine can have a crucial effect on keeping levels of odorants during aging, and therefore, in wine shelf-life. Within the latter, some of the odorants accumulating during aging, such as DMS, TDN, or TPB, may have controversial effects on wine quality, and may therefore have also a major influence on wine longevity.

For of all these reasons, the control of grape-derived wine aroma is an essential piece for controlling wine quality and wine shelf-life. Comprehensive analytical strategies for such a control have to face demanding challenges, which at present are not satisfactorily solved. On the one hand, aroma molecules with different chemophysical properties have to be simultaneously determined, which is nearly impossible using a single isolation strategy. On the other hand, the strategy has to sort out the difficult and non-obvious link between specific precursors and wine odorants. Surely this will require combining metabolomic approaches with new, comprehensive hydrolytical strategies. Both techniques are at hand but will require from researchers a clear awareness of all the dimensions of the analytical problem.

## Figures and Tables

**Table 1:** Structures, odor properties, and occurrence of the key odorants of aromatic grapes.

Compound	Structure	Grape	Odor Description	Threshold	Range of Occurrence in Grapes
Linalool	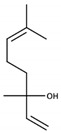	Muscat	Hyacinth, Muscat wine	6 μg/L [48]	0.06–1.5 mg/L [28]
Geraniol	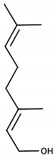	Muscat	Citrus, rose	40 μg/L [49]	0.09–1.1 mg/L [28]
(Z)-Rose oxide	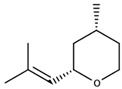	Traminer	Rose, litchi	0.5 (l form) or 50 μg/L (d form) [34]	7–29 μg/L [35]
o-Aminoacetophenone	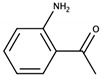	Concord	Sweet, caramel	0.2 μg/L [50]	10–20 μg/L [46]
Methyl anthranilate	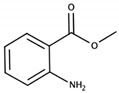	Concord	Orangine, sweet	3 μg/L [51]	0.8 mg/kg [39] 0.5–6 mg/kg [52]

**Table 2:** Structures, odor properties, and occurrence of norisoprenoids found above their threshold value in wine.

Compound	Structure	Odor Descriptor	Threshold in Wine	Range of Occurrence in Wine
β-Damascenone	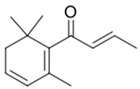	Plum, cooked apple	50 ng/L [32]	n.d. to 10.5 μg/L [71]
β-Ionone	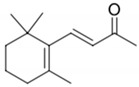	Violet, woody	90 ng/L [72]	n.d. to 1.2 μg/L [71]
TDN	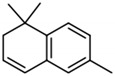	Kerosene-like	2 μg/L [73]	n.d. to 255 μg/L [74]
TPB	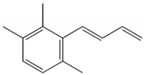	Green, cut-grass	40 ng/L [75]	n.d. to 233 ng/L [76]

n.d.: Not detected.

**Table 3:** Structures, odor properties, and occurrence of alkylmethoxypyrazines.

Compound	Structure	Odor Descriptor	Odor Threshold	Range of Occurrence in Grape Juice
3-Isobutyl-2-methoxypyrazine	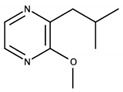	Bell pepper, earthy	2 ng/L (in water) [103]; 15 ng/L (in wine) [94]	n.d. to 79 ng/L [93]
3-Isopropyl-2-methoxypyrazine	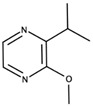	Green pea, earthy	0.74–1.11 (hybrid grape juice) [104]; 2 ng/L in wine [105]	n.d. to 6.8 ng/L [93]
3-Secbutyl-2-methoxypyrazine	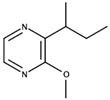	Bell pepper	1–2 ng/L (in water) [106]	n.d. to 1.3 ng/L [93]

n.d.: Not detected.

**Table 4:** Structures, odor properties, and occurrence of FAO-related ^1^ family of compounds.

Compound	Structure	Odor Descriptor	Threshold in Water	Ranges of Occurrence in Grape [23,25,57,61,110,111,112]
Hexanal	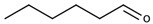	Herbaceous	5 μg/L [113]	8–1300 μg/kg
(Z)-3-Hexenal	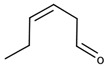	Grass	0.25 μg/L [48]	4–20 μg/kg
(E)-2-Hexenal	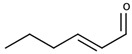	Grass	17 μg/L [113]	13–3800 μg/kg
(E,E)-2,4-Hexadienal	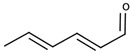	Grass	60 μg/L [114]	50–120 μg/kg
(Z)-3-Hexenol	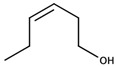	Grass	70 μg/L [48]	4–79 μg/kg
(E)-2-Hexenol	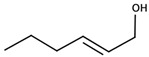	Green	400 μg/L [114]	
1-Hexanol	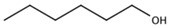	Green	2500 μg/L [113]	45–214 μg/kg
E-2-Nonenal	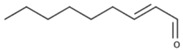	Green, fatty	0.17 μg/L [113]	
(E,Z)-2,6-Nonadienal	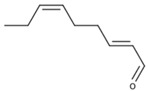	Cucumber	0.01 μg/L [115]	113–482 μg/kg

^1^ FAO: Fatty acid oxidation.

**Table 5:** Wine norisoprenoid and terpene odorants coming from specific precursors.

Aroma Molecule	Enzymatic Hydrolysis	Harsh Acid Hydrolysis	Mild/Long Term Acid Hydrolysis
**Norisoprenoids**			
β-Damascenone	Not found; yes in raisins [23,57] and frozen grapes [136]; not in wines [137]; 0.17–0.5 ppb in frozen grapes [12]	26 ppb [138]; detected by GCO [139]; 4–28 ppb depending varieties, unclear pulp/skin distribution [140]; 4–20 ppb depending location [140]; levels correlated to total norisoprenoids by enzymatic [141]; 2–4.5 ppb depending varieties [12]	Detected by GCO [142]; maxima (3.3 ppb) after short aging, then steady decrease [14]; steady increase all the aging in fermented samples [143]; maxima 7.1–7.3 ppb after medium aging in unfermented controls [143]; formed soon and stable, maxima 17 ppb [15]; idem, with maxima 7 ppb [66]
β-Ionone	Not found; yes in frozen grapes [136]; not in wines [137]; <0.11 ppb in frozen grapes [12]	Generally yes; not found in [12]	Maxima (1.9 ppb) after short aging, stable with time [14]; formed soon, stable for a while, maxima 7.7 ppb [15]
TDN	Not found; yes in frozen grapes [136]; not in wines [137]; 1–6 ppb (5–30% of levels found in harsh acid hydrolysis) in frozen grapes [12]	8 ppb [138]; detected by GCO [139]; 1–35 ppb depending on varieties, unclear pulp/skin distribution [140]; n.d. to 26 ppb depending on place [140]; 8–89 ppb depending on varieties [12]	Linear increase with time, max 140 ppb [143]; idem, max at 61 ppb [15]; idem [66]
TPB	Not found; 0.2–3 ppb (2–22% of levels found in harsh acid hydrolysis) in frozen grapes [12]	3 ppb [138]; 2–23 ppb depending varieties [12]	Continuously formed, maxima 9 ppb [66]
**Terpenes**			
Linalool	Generally present; not found in Portuguese reds [140]; not found in Melon B [141]; not found in Shiraz [144]; found at low levels (less than 7% geraniol 1% total terpenes) [144]	3% levels found in enzymatic [138]; 10–50% of levels found in enzymatic [12]	Found only in mild acid hydrolysis [141]; maxima after fermentation, sharp decrease in aging [14]; in Grenache, maxima after short aging [143]; formed very soon, sharp decrease [15,66]
Geraniol	Always found; up to 10% of total terpenes in Shiraz, 14% in Muscat [144]	No [138]; 3–30% of levels found in enzymatic [12]	Maxima in fermentation, sharp decrease in aging [14,143]; formed very soon, sharp decrease [15,66]
(Z)-Rose oxide	11–29 ppb in Muscat, depending on maturity [145]; unrelated to free form in raisins [23]	0.04 ppb in Muscat, 0.01 ppb in Grenache; not found in Verdejo, Tempranillo, Chardonnay, Cabernet Sauvignon, or Merlot [12]	
Geranic acid	Up to 2–3 ppm [146,147]; also found in raisins [23]; <4 ppb [145]; up to 7.5% total terpenes in Shiraz, 18% in Muscat [144]	Not found [138]; 0.5–50% of levels found in enzymatic [12]	1.5 ppb in Chardonnay juices [148]
Piperitone			Derived from limonene, unknown accumulation pattern [149]; limonene accumulates in the first periods of aging, then slight decrease [66]

n.d.: Not detected.

**Table 6:** Wine benzenoid odorants coming from specific precursors.

Aroma Molecule	Enzymatic Hydrolysis	Harsh Acid Hydrolysis	Mild/Long Term Acid Hydrolysis
**Volatile Phenols**			
Guaiacol	Not found [146]: only in Brachetto, not in Aleatico, Malvasia, or Moscato [147]; <2 ppb [125]; up to 60 ppb in Rojal wine [137]; 0–41 ppb [150]; 10–76 ppb depending on vintage [151]; 15–44 ppb depending on vintage [152]; 17 ppb in Shiraz [144]; 0.4–2.3 ppb depending on varieties [12]	Detected by GCO [139]; <0.61 ppb, unrelated to enzymatic levels [12]	Detected by GCO [142]; Steady increase with time, maxima 4.3 ppb [14]; idem, maxima 6.3 ppb [143]; idem, maxima 14 ppb [15]
Eugenol	1–8.3 ppb [146,147]; not found [125]; up to 33 ppb in Rojal wine [137]; present in less than half varieties, up to 16 ppb [150]; 84–216 ppb depending on vintage [151]; 12–20 ppb in Bobal depending on vintage [152]; n.d. to 9.4 ppb depending on variety [140]; 2.7–18 ppb depending on location [140]; 10 ppb in Shiraz [144]; 0.4–7 ppb depending on variety [12]	Detected by GCO [139]; <0.36 ppb, unrelated to enzymatic levels [12]	Steady increase, maxima 1.25 ppb [15]
Isoeugenol	Up to 14 ppb in Rojal wine [137]; 7.6–26 ppb depending on vintage [151]; 5–25 ppb depending on vintage [152]; 0.4–4.8 ppb depending on varieties [12]	<0.58 ppb, unrelated to enzymatic levels [12]	Detected by GCO [142]
2,6-Dimethoxyphenol	3–60 ppb [147]; n.d. to 13 ppb depending on varieties [12]	n.d. to 5.5 ppb depending on varieties [12]	Detected by GCO [142]; steady increase with time, maxima 33 ppb [14]; idem, maxima 142 ppb [15]
4-Vinylguaiacol	65–357 ppb [147]; <24 ppb [150]; 56–378 ppb depending on vintage [151]; 56–64 ppb depending on vintage in Bobal [152]; 2–114 ppb depending on varieties [140]; 2–178 ppb depending on location [140]; 21 ppb in Shiraz [144]; 39–162 ppb on depending varieties [12]	40% of enzymatic [138]; detected by GCO [139]; 10–38 ppb depending on varieties, unrelated to enzymatic [12]	A maxima (21 ppb) after short aging, then decrease and steady increase [14]; continuous increase, maxima 5.5 ppm [143]; formed soon and stable, maxima at 1.3 ppm [15]
4-Vinylphenol	28–266 ppb [150]; 5–222 ppb depending on varieties [140]; 19–310 ppb depending on location [140]; 6 ppb in Shiraz [144]; 121–1739 ppb depending on varieties [12]	9–21 ppb depending on varieties, unrelated to enzymatic [12]	A maxima after short aging (45 ppb), then decrease and steady increase, maxima 80 ppb [14]; continuous increase, maxima 4.4 ppm [143]; formed very soon, later steady decrease, maxima at 102 ppb [15]
**Vanillin Derivatives**			
Vanillin	27–42 ppb [147]; 361 ppb in skin of Uva di Troia [125]; 31–61 ppb [137]; <37 ppb [150]; 48–68 ppb depending on vintage [151]; 60–160 ppb depending on vintage in Bobal [152]; 31 ppb in Shiraz [144]; 40 ppb in Muscat [144]; <4.1 ppb [12]	50% enzymatic [138]; detected by GCO [139]; <1.5 ppb [12]	Detected by GCO [142]; linear increase with time, maxima 45 ppb [14]; idem, maxima 91 ppb [143]; idem, maxima 123 ppb [15]
Methyl vanillate	4–7 ppb [147]; <7 ppb [125]; up to 205 ppb in Rojal wine [137]; <42 ppb [150]; 12–147 ppb depending on vintage [151]; 9–143 depending on vintage in Bobal [152]; 25 ppb in Shiraz [144]; 154 ppb in Muscat [144]; <18 ppb [12]	<3.4 ppb [12]	6 ppb in Chardonnay juices [148]
Ethyl vanillate	Up to 45 ppb in Rojal wine [137]; n.d. to 10 ppb depending on vintage in Bobal [152]; <12 ppb [12]	<3.1 ppb	
Acetovanillone	Up to 205 and 260 ppb in Rojal and Tortosí wines [137]; 1–12 ppb depending on vintage [151]; 42 ppb in Muscat, none in Shiraz [144]; 8–34 ppb depending on variety [12]	Detected by GCO [139]; <2.5 ppb, unrelated to enzymatic [12]	Unclear pattern [15]; 5 ppb in Chardonnay juices [148]
**Cinnamic Acid Derivatives**			
Ethyl cinnamate	7 ppb only in pulp from Uva di Troia [125]; <0.8 ppb [12]; its precursor, cinnamic acid has been found up to 7 ppb in fractions from wine, levels depending on vintage [137,151,152]	12 ppb [138]; <0.11 ppb [12]	Detected by GCO [142] [15]; steady increase with time in some varietals, maxima 3.3 ppb [14]; maxima 3.3 ppb after short aging [143]

n.d.: Not detected.

**Table 7:** Wine miscellaneous odorants coming from specific precursors.

Aroma Molecule	Enzymatic Hydrolysis	Harsh Acid Hydrolysis	Mild/Long Term Acid Hydrolysis
Ethyl cyclohexanoate			Its precursor, ethyl cyclohexanoic acid, found in unfermented mistellas [153]
Ethyl 4-methylpentanoate			Its precursor, ethyl 4-methylpentanoic acid, found in unfermented mistellas [153]
γ-Decalactone	No [125]	Identified [8]	Detected by GCO [15,142]
Massoia lactone			Detected by GCO [15]
Furaneol	Aglianico up to 2 ppm in pulp and 0.6 in skin, Uva di Troia 1,2 ppm in pulp, 90 ppb in skin [125]; 15–51 ppm in muscadine [46]	Detected by GCO [139]	Detected by GCO [15]
DMS			Only found in grape or grape mistellas not in precursor fractions [15]
**Polyfunctional Mercaptans**			
4-Methyl-4-mercaptopentan-2-one			Mostly released by yeast.
3-Mercaptohexanol			Released by yeast. Detected by GCO in mild-acid hydrolyzates [15,142]
3-Mercaptohexyl acetate			Formed by yeast from 3MH

**Table 8:** Structures, odor properties, and occurrence of varietal thiols.

Compound	Structure	Odor Descriptor	Threshold in Model Wine (ng/L) [188]	Range of Occurrence in Wine (ng/L) [189]
4-Methyl-4-mercaptopentan-2-one	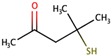	Box tree	0.8	n.d. to 90
3-Mercaptohexanol	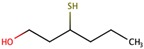	Grapefruit	60	n.d. to 7300
3-Mercaptohexyl acetate	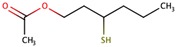	Box tree, passion fruit	4	n.d. to 440

n.d.: Not detected.

**Table 9:** Mean concentration of 4MMP and 3MH cysteinylated and gluthanionylated precursors in μg/L ± RSD% (n = 2) in eight grape varieties [197].

Variety	CYS–MH	CYS–MMP	GLU–MH	GLU–MMP
Sauvignon Blanc	174 ± 7	12.6 ± 1.4	1557 ± 86	7.7 ± 1.3
Gewürztraminer	89 ± 6	8.0 ± 1.5	1154 ± 56	6.6 ± 0.8
Muscat	157 ± 8	n.d.	1673 ± 71	8.3 ± 0.9
Grenache	172 ± 5	7.9 ± 1.2	1422 ± 63	9.4 ± 1.2
Albariño	158 ± 3	7.2 ± 0.7	1462 ± 80	8.4 ± 0.7
Tempranillo	205 ± 8	6.1 ± 1.8	1284 ± 76	10.3 ± 1.1
Verdejo	215 ± 9	7.3 ± 1.0	3397 ± 102	n.d.
Chardonnay	32 ± 4	0.4 ± 0.2	1405 ± 97	n.d.

n.d.: Not detected.
